# How does exercise regulate the physiological responses of post traumatic stress disorder? the crosstalk between oxidative stress and the hypothalamic-pituitary-adrenal axis

**DOI:** 10.3389/fphys.2025.1567603

**Published:** 2025-09-12

**Authors:** Tianyu Zhang, Jianda Kong

**Affiliations:** ^1^ School of Psychology, Qufu Normal University, Qufu, Shandong, China; ^2^ College of Sports Science, Qufu Normal University, Qufu, Shandong, China

**Keywords:** post traumatic stress disorder, exercise, oxidative stress, hypothalamic-pituitary-adrenal axis, antioxidant defense, aerobic exercise, strength training

## Abstract

Post traumatic stress disorder (PTSD) is a severe psychological disorder cautilized by extreme traumatic events, typically accompanied by physiological mechanisms, such as oxidative stress and dysfunction of the hypothalamic-pituitary-adrenal (HPA) axis. Our review explores how exercise boosts the physiological response of PTSD patients by regulating oxidative stress and HPA axis function, and delves into the potential mechanisms and application prospects of exercise in PTSD treatment. By a review of existing literature, we mainly discussed the effects of various types of exercise, like aerobic exercise, strength training, and high-intensity interval training (HIIT), on oxidative stress markers and HPA axis function, and evaluated the efficacy and mechanism of exercise intervention in the treatment of post traumatic stress disorder. Specifically, regular exercise can enhance the antioxidant defense system, reduce levels of reactive oxygen species (ROS), restore HPA axis function, and thereby alleviate the psychological and physiological symptoms of post-traumatic stress disorder. Different types of exercise have varying influences on the regulation of oxidative stress and cortisol secretion patterns. As a multidimensional therapeutic approach, exercise can provide comprehensive therapeutic advantages by enhancing neural plasticity, promoting immune function, and improving psychological resilience. In addition, the combination of exercise with conventional treatment strategies, such as cognitive-behavioral therapy and medication has apparently optimized treatment outcomes.

## 1 Introduction

Post Traumatic Stress Disorder (PTSD) is a severe psychological disorder induced by an individual’s experience of extreme psychological trauma, generally displayed as flashbacks, nightmares, emotional numbness, avoidance behaviors, coupled with anxiety and irritability (WHO). In the context of data from the World Health Organization (WHO), near 3.9% of adults globally have experienced PTSD (WHO). Literature has shown that external traumatic causes, like war, natural disasters, and violent events are the major factors of PTSD (Koenen et al., 2017). The incidence of PTSD is vital among specific populations, involving military personnel, emergency healthcare workers, and survivors of natural disasters (Koenen et al., 2017). PTSD, what is worth noting is that, not only affects patients’ mental wellbeing and quality of life, but may also lead to a range of physical health problems, such as cardiovascular diseases and immune dysfunction (Koenen et al., 2017). Therefore, PTSD has developed into a major global challenge in the field of mental health, calling for the continuous exploration of new treatment strategies. Though conventional pharmacological and psychological therapies have been totally applied in the intervention of PTSD patients, they still have some limitations (Björkman and Ekblom, 2022; Hegberg et al., 2019a).

Exercise has been regarded as an efficacious adjunctive treatment by degress (Hegberg et al., 2019a; Jadhakhan et al., 2022). Articles have displayed that moderate exercise can not only apparently boost mental health levels, but also slow down emotional symptoms, like anxiety and depression, which are important for PTSD patients (Hegberg et al., 2019a; Jadhakhan et al., 2022). Moreover, exercise further facilitates individuals’ adaptability and stress resilience by activating a variety of physiological mechanisms, like the release of endorphins and the regulation of inflammatory responses (Björkman and Ekblom, 2022; Jadhakhan et al., 2022). Although the potential of exercise in slowing down PTSD symptoms is gradually being determined, how exercise boosts the physiological responses of PTSD by regulating oxidative stress and the hypothalamic-pituitary-adrenal (HPA) axis continues to be a comparatively new research area (Hegberg et al., 2019a). Recently, evidence has demonstrated that oxidative stress and HPA axis dysfunction are crucial physiological mechanisms in PTSD (Karanikas et al., 2021). It is worth noting that increased oxidative stress and HPA axis dysregulation may contribute to abnormal stress responses in individuals, playing a vital role in the occurrence and maintenance of PTSD (Pinna et al., 2023). Exercise may have vital impact on the physiological responses of PTSD by increasing oxidative stress responses and regulating HPA axis function, thus offering a new direction for PTSD treatment (Nowacka-Chmielewska et al., 2022). As a consequence, an in-depth exploration of how exercise affects its therapeutic potential at these physiological levels, in particular concerning the mechanisms of oxidative stress and the HPA axis, is vital for a deeper insight of the potential of exercise in PTSD treatment.

This review centers on investigating the mechanisms by which exercise manages PTSD through the regulation of oxidative stress and the HPA axis. By investigating existing literature, our review seeks to provide a theoretical foundation for future research and offer new treatment thoughts for clinical practice, thus promoting new progressions in PTSD treatment.

## 2 Physiological influences of exercise on post traumatic stress disorder

### 2.1 Influences of exercise on the nervous system

The effects of exercise on brain structure and function have been completely studied, in detail, in brain regions related to emotion regulation and stress response, like the amygdala and prefrontal cortex (Ferrer-Uris et al., 2022). Study has illustrated that regular physical exercise can promote brain plasticity, boost cognitive function, and affect emotional states (Cabral et al., 2019). For instance, exercise can boost levels of neurotrophic factors (like brain-derived neurotrophic factor, BDNF), promoting the growth and survival of neurons, thus enhancing brain structure and function (Liang et al., 2021).

When it comes to emotion regulation, exercise has been proved to vastly affect the levels of neurotransmitters related to emotions and stress responses, like dopamine and norepinephrine (Marques et al., 2021). Exercise can activate the brain’s reward system, increasing the release of these neurotransmitters, thus contributing to mood and lowering anxiety (Chen et al., 2017). Furthermore, exercise can lower cortisol levels relared to pressure, thus alleviating the disadvantageous effects of chronic stress on the brain (Chen et al., 2017). In particular, the influences of exercise on the prefrontal cortex and amygdala are quite vital The prefrontal cortex plays a critical role in executive functions and emotion regulation, while the amygdala is closely linked to emotional responses and fear memory (Maurer et al., 2022). Studies have dispalyed that regular exercise can promote the function of the prefrontal cortex, boost individuals’ emotion regulation abilities, and lower the overactivity of the amygdala, thus assisting in individuals better cope with pressure and anxiety (Zhao et al., 2020; van der Stouwe et al., 2018). In addition, exercise is viewed to boost overall brain health by promoting cerebral blood circulation and neural connectivity (Cabral et al., 2019; Liang et al., 2021). Particularly, exercise can strengthen cerebral blood flow, boosting the supply of oxygen and nutrients to the brain, thus facilitating neuronal function and survival (Cabral et al., 2019; Liang et al., 2021). This enhancement not only leads to cognitive function enhancement, but may also play an vital role in the prevention and treatment of mental health issues, like depression (Zhao et al., 2020; Eyre and Baune, 2012).

To sum up, the effects of exercise on the nervous system are multifaceted, including brain structure, function, and neurotransmitter regulation. By enhancing neural plasticity and boosting emotion regulation, exercise supplies crucial support for mental health.

### 2.2 Influences of exercise on the immune system

Exercise is totally known as an efficacious intervention approach that can boost the immune function and inflammatory responses in PTSD patients (Hegberg et al., 2019a). Study has displayed that PTSD patients generally go along with systemic inflammatory responses, which are closely related to a variety of physiological and psychological health problems (Pace and Heim, 2011). Exercise regulates inflammatory markers by multiple mechanisms, thus promoting immune homeostasis (Scheffer and Latini, 2020).

Exercise can vastly lower inflammation markers linked to PTSD (Hegberg et al., 2019a). Acute exercise can promote endogenous anti-inflammatory responses, contributing to a reduction in the generation of pro-inflammatory cytokines, like tumor necrosis factor-alpha (TNF-α) (Dimitrov et al., 2017). In one evidence, interestingly, it was demonstrated that moderate-intensity exercise could inhibit TNF production by monocytes through β2-adrenergic receptors, emphasizing that exercise can regulate immune responses by activating the sympathetic nervous system (Dimitrov et al., 2017). This mechanism may be crucial in PTSD patients, whose immune systems are commonly in a hyper-reactive state (Dimitrov et al., 2017). Exercise can also indirectly affect inflammatory responses by enhancing mental health. Literature has illustrated that exercise can slow down PTSD symptoms, thus lowering inflammation levels relato psychological pressure (Pace and Heim, 2011). For instance, exercise can enhance neuroprotection and anti-inflammatory influences, boosting the neuroimmune environment in the brain, thus helping with slowing down emotional disorders, like depression and anxiety, which are commonly seen in PTSD patients (Pace and Heim, 2011). In addition, exercise may affect immune function through regulating metabolic pathways. Exercise can promote the metabolism of tryptophan in muscles, enhancing the generation of kynurenine, which plays an vital role in regulating immune responses and energy homeostasis (Martin et al., 2020). These metabolic changes may help boost the thorough health status of PTSD patients, lowering the risk of chronic inflammation (Rangel et al., 2025). Therefore, exercise regulates inflammation linked to PTSD through multiple mechanisms, promoting immune homeostasis.

Generally speaking, exercise is an efficacious approach for enhancing immune function and decreasing inflammation in PTSD patients. It lowers inflammation markers by enhancing anti-inflammatory responses and decreasing pro-inflammatory cytokines like TNF-α. Exercise also slows down PTSD symptoms, thus helping mental wellbeing and decreasing inflammation related to psychological pressure. Moreover, exercise regulates metabolic pathways, like tryptophan metabolism, to support immune function and energy homeostasis. These combined effects reduce chronic inflammation and improve overall health in PTSD patients.

## 3 Oxidative stress and the role of exercise in PTSD modulation

**FIGURE 1:**
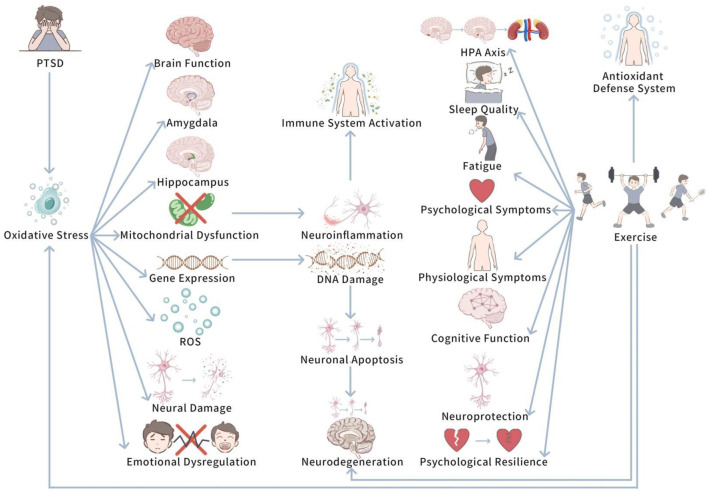
The role of oxidative stress in PTSD and the modulatory influence of exercise.

Oxidative pressure plays a pivotal role in determining the potential biological mechanisms of PTSD. Upon in-depth investigation, we further delve into how oxidative pressure specifically influences PTSD at the cellular and neurological levels. Figure 1 *shows Oxidative Stress and the Modulatory Role of Exercise in PTSD.*

This diagram illustrates how PTSD-induced oxidative pressure influences brain regions (e.g., amygdala and hippocampus) and leads to neuroinflammation, mitochondrial dysfunction, and cognitive decline. It also shows how exercise can mitigate oxidative stress by enhancing antioxidant defenses, thus reducing neuroinflammation and promoting cognitive recovery.

### 3.1 The Role of Oxidative Stress in PTSD

PTSD has been unfolded to be closely related to oxidative pressure, in particular in terms of neural damage and brain function hinderment (Kim TD. et al., 2020). At length, PTSD patients generally experience prolonged pressure and traumatic experience, which trigger changes in the function of the nervous system and deepen oxidative stress responses (Kim TD. et al., 2020). Studies have displayed that in PTSD patients, levels of reactive oxygen species (ROS) are commonly augmented in the brain and peripheral tissues, and the function of the antioxidant defense system is weakened (Kim TD. et al., 2020; Lushchak et al., 2023). These changes in oxidative pressure levels trigger cellular damage in the brain, involving neurons, in particular,in brain regions closely related to emotion and memory, like the amygdala and hippocampus (Lushchak et al., 2023; Zheng et al., 2022). In PTSD research, interestingly, oxidative pressure is seen to play a vital role in the disease’s progression (Pinna et al., 2023; Fesharaki-Zadeh, 2022). Oxidative pressure boosts the generation of ROS, triggers lipid peroxidation of cell membranes, thus hindering cell function and structure (Pinna et al., 2023; Fesharaki-Zadeh, 2022). At the same time, the accumulation of ROS can disrupt mitochondrial function, leading to mitochondrial damage, which is important for neural plasticity and cognitive function in the brain (Pinna et al., 2023). Mitochondrial dysfunction is closely related to the exacerbation of neuroinflammatory responses, which further enhances the overactivation of the immune system, developing a vicious cycle (Fesharaki-Zadeh, 2022; Franzoni et al., 2021). In addtion, high levels of ROS can affect gene expression by inducing oxidative damage to DNA, augmenting neuronal apoptosis and neurodegeneration (Fesharaki-Zadeh, 2022). These biochemical changes not only impact brain structure, but may also trigger long-term dysregulation of emotion regulation mechanisms, making it hard for patients to recover from traumatic experience (Pinna et al., 2023; Franzoni et al., 2021).

To sum up, PTSD is closely linked to oxidative pressure, which facilitates neural damage and brain function impairment. Prolonged pressure and trauma elevate reactive oxygen species (ROS) levels in PTSD patients, harming antioxidant defense systems and triggering cellular damage, especially in the amygdala and hippocampus. This oxidative stress disrupts cell function, damages mitochondria, and worsens neuroinflammation, creating a cycle that deepens the disorder. In addition, ROS-induced DNA damage and increased neuronal apoptosis can lead to long-term emotional dysregulation and prevent recovery.

### 3.2 The impact of exercise on oxidative stress

#### 3.2.1 Exercise and the antioxidant defense system

Regular physical exercise, especially aerobic exercise, can lower the generation of free radicals by adding the activity of antioxidant enzymes, thus slowing down oxidative damage (He et al., 2016). Studies have demonstrated that aerobic exercise can increase the activity of superoxide dismutase (SOD), catalase (CAT), and glutathione peroxidase (GPx) (Meng and Su, 2024; Awang et al., 2022). These enzymes help maintain cellular redox balance by scavenging ROS in the body, reducing damage caused by oxidative pressure (Awang et al., 2022). Moderate-intensity aerobic exercise, obviously, like running and swimming, has been displayed to vastly boost the activity of antioxidant enzymes (Thirupathi et al., 2021a; Börzsei et al., 2024). In particular, long-term endurance training can boost the activity of antioxidant enzymes in skeletal muscle, like SOD, CAT, and GPx, further lowering exercise-induced oxidative pressure (Powers et al., 2022). These studies prove that proper aerobic exercise not only boost the activity of antioxidant enzymes, but also counterplays the oxidative pressure induced by work-out, enhancing the body’s antioxidant defense system.

To sum up, aerobic exercise, particularly moderate-intensity activities like running and swimming, facilitates the activity of antioxidant enzymes (SOD, CAT, and GPx), helping to alleviate oxidative pressure and remain cellular redox balance. Long-term endurance training promotes these enzymes in skeletal muscle, promoting the body’s antioxidant defense and reducing exercise-induced oxidative damage.

#### 3.2.2 The impact of exercise type on oxidative stress

The affect of exercise type on oxidative pressure is a complicated physiological process comprising various regulatory mechanisms (Meng and Su, 2024). Multiple typesvarious types of exercise, like aerobic exercise, strength training, and high-intensity interval training (HIIT), affect the body’s redox balance and regulate the activity of the antioxidant defense system in their unique ways, thus affecting oxidative stress to varying degrees (Meng and Su, 2024).

Aerobic exercises (such as running, cycling, etc.) are regarded as a way to enhance antioxidant capability and lower oxidative damage by improving blood circulation and oxygen supply, boosting cellular energy metabolism (Meng and Su, 2024). A vital role of aerobic exercise is to activate the body’s antioxidant enzyme systems, contributing to slow down oxidative stress caused by exercise (So et al., 2022). For instance, moderate aerobic exercise has been displayed to strike a balance between free radical generation and antioxidant responses, reducing oxidative damage (Kawamura and Muraoka, 2018). By augmenting oxygen supply, aerobic exercise not only boosts cellular energy generation, but also enhances antioxidant substances in the blood, thus improving the body’s ability to cope with oxidative stress (Vargas-Mendoza et al., 2021). This makes aerobic exercise an efficacious approach of preventing and slowing down oxidative damage, in particular in long-term or sustained exercise practices, which can much more vastly improve antioxidant defense capacity (Moir et al., 2023).

The role of strength training is somehow different from aerobic exercise. Though its direct impact on oxidative stress is comparatively a little small, strength training can still boost the antioxidant system (Thirupathi et al., 2021b). Strength training induces the generation of oxidative stress by raising muscle oxygen consumption and metabolic load (Powers et al., 2022). Nevertheless, this process meanwhile activates the body’s antioxidant system to handle the generated oxidative stress (Kawamura and Muraoka, 2018). Strength training triggers adaptive changes in muscles, improving muscle cells’ tolerance to oxidative damage, thus helping to enhance antioxidant defense capacity (Meng and Su, 2024). Long-term strength training helps to stimulate the activity of antioxidant enzymes in the body, particularly in high-intensity training and high-load exercise projects, where the expression of antioxidant enzymes is more pronounced (Powers et al., 2022).

HIIT is a training strategy that integrate short bursts of high-intensity exercise with periods of low-intensity recovery. Its unique training pattern can obviously boost the activity of antioxidant enzymes and produce beneficial regulatory influences on the nervous system (Dos Santos et al., 2020). Though HIIT training initially boosts short-term oxidative stress responses, as training progresses, the body’s antioxidant ability is vastly enhanced (Meng and Su, 2024). HIIT has a apparently influence on boosting exercise capacity and lowering fatigue induced by oxidative stres (Meng and Su, 2024). Studies have demonstrated that HIIT can not only enhance the activity of antioxidant enzymes, but also boost antioxidant capacity, further improving overall exercise performance and recovery speed (Moir et al., 2023). Moreover, HIIT can remarkably enhance antioxidant status in populations without training experience (Lu et al., 2021).

In general, diverse kinds of exercise regulate oxidative stress levels in the body through different mechanisms, and their influences vary when it comes to exercise intensity, modality, and individual adaptability. Whether aerobic exercise, strength training, or HIIT, they can all boost the enhancement of antioxidant capacity to some extent and alleviate the oxidative damage generated in the course of exercise. Nevertheless, the choice of exercise type should be thoroughly considered based on individual training goals, physical fitness levels, and health conditions to achieve the optimal antioxidant influence.

### 3.3 The association between oxidative stress and PTSD symptoms

Exercise may influence the physiological and psychological symptoms of PTSD by regulating oxidative stress (Rosenbaum et al., 2015). PTSD patients commonly exhibit emotional dysregulation (such as anxiety, depression, irritability) and physiological symptoms (such as sleep disruptances and fatigue), and oxidative stress is viewed as an important biological mechanism triggering these symptoms (Rosenbaum et al., 2015).

Research has illustrated that increased levels of oxidative stress are closely linked to anxiety and depression symptoms in PTSD patients (Liu et al., 2015). Too much oxidative damage can damage neurotransmitter systems, especially neurotransmitters like dopamine and norepinephrine, which play vital roles in emotion regulation (Lushchak et al., 2023). Exercise, by lowering reactive oxygen species (ROS) levels, helps maintain neurotransmitter balance, thus slowing down emotional symptoms, such as anxiety and depression (Nowacka-Chmielewska et al., 2022).

Common physiological symptoms in PTSD patients, such as poor sleep quality and increased fatigue, are closely linked to oxidative stress (Zhang et al., 2024). Oxidative stress reactions, particularly the excessive generation of reactive oxygen species (ROS), may interfere with the function of the hypothalamic-pituitary-adrenal (HPA) axis, leading to hormonal imbalances that impact sleep and physical stamina (Spiers et al., 2015). Studies have demonstrated that the HPA axis in PTSD patients is general in an abnormally hyperactive state, which may cause insomnia and fatigue. A ystematic review and meta-analyses by Björkman et al. further confirm this (Björkman and Ekblom, 2022). Oxidative stress is not only related to neuroinflammatory responses, but also impacts the functional regulation of stress-responsive brain regions (Joëls, 2008). This mechanism may be a main factor triggering physiological dysfunction in PTSD patients. Björkman et al. also observed that exercise, as an efficacious intervention, can help boost oxidative stress levels and slow down abnormal HPA axis activity, thus promoting sleep quality and lowering fatigue (Björkman and Ekblom, 2022). Hence, regular physical exercise can not only slow down the psychological symptoms of PTSD, but also apparently stimulate patients’ physiological symptoms by regulating oxidative stress and restoring normal HPA axis function.

Exercise has an apparent promoting influence on the physiological and psychological symptoms of PTSD patients, particularly in regulating oxidative stress, with aerobic exercise showing more prominent influences (Meng and Su, 2024). Multiple studies have demonstrated that aerobic exercise not only slows down anxiety and depression symptoms in PTSD patients, but also apparently boosts sleep quality and daily functioning (Meng and Su, 2024). This finding displays that exercise has an impact on a positive effect on PTSD treatment by slowing down emotional disorders and promoting individuals’ resilience. Another vital role of exercise is to facilitate antioxidant capacity, providing protection for the nervous system. By regulating oxidative stress responses, exercise can enhance the body’s antioxidant capacity, slow downing the progression of neurodegenerative changes (Bacanoiu et al., 2023). This mechanism is not only advantageous for the health of the nervous system, but also facilitate cognitive function in PTSD patients (Xie et al., 2021). Studies have demonstrated that moderate exercise can lower oxidative damage in the brain, thus protecting neurons from stress and degenerative changes (Nowacka-Chmielewska et al., 2022). Moreover, the long-term strengths of exercise for PTSD patients are not limited to promoting antioxidant capacity, but also comprise increasing the brain’s adaptability to stress, thus promoting individual psychological resilience (Meng and Su, 2024). This displays that exercise can supply sustained psychological health enhancement and recovery opportunities for PTSD patients.

Generally speaking, relevant studies clearly prove that exercise can slow down PTSD symptoms and supply neuroprotection through multiple physiological mechanisms, particularly by antioxidant influences, while also promoting patients’ cognitive and emotional states. As an adjunctive treatment for PTSD, exercise has displayed tremendous potential and practical influences, worthy of further promotion in clinical practice.

### 3.4 Comparing findings from animal models and human studies

The exploration of the influences of exercise on PTSD has been widely studied in both animal models and human populations, uncovering comprehensive insights into the mechanisms and therapeutic potential of physical activity. While animal studies offer controlled environments for examining biological and physiological processes, human studies offer more complicated and ecologically valid data. Here, we synthesize findings from both domains to highlight key differences, compare results, and reflect on the implications for PTSD treatment.

Human studies, like those by Browne et al. (2021) (Browne et al., 2021) and Hall et al. (2020) (Hall et al., 2020), focus on the effects of exercise interventions on PTSD symptoms and relevant health outcomes in older veterans. Browne et al. (2021) (Browne et al., 2021) and Hall et al. (2020) found that a 12-week supervised exercise intervention did not apparently improve diet quality, despite advantageous effects on physical wellbeing, emphasizing the challenges of simultaneously addressing both psychological and physical health outcomes in PTSD populations. In contrast, studies like those of Powers et al. (2015) (Powers et al., 2015) and Young-McCaughan et al. (2022) found that while exercise, such as aerobic training or exercise augmented exposure therapy, can promote PTSD symptoms in military personnel and veterans, the efficacy of combining exercise with therapeutic interventions (e.g., imaginal exposure) may not outperform the impact of exercise alone. These studies suggest that while exercise can alleviate symptoms such as anxiety and depression, the extra advantages of integrating exercise with exposure therapy are not always clear-cut.

Animal models have been pivotal in uncovering the physiological underpinnings of PTSD and the mechanisms by which exercise regulates these processes. Studies like those by Manukhina et al. (2021) and Koyuncuoğlu et al. (2021) demonstrate that exercise can apparently regulate oxidative stress, inflammation, and cognitive function in PTSD. For instance, Koyuncuoğlu et al. (2021) demonstrated that HIIT in rats exposed to PTSD through a single prolonged stress (SPS) protocol mitigated cognitive decline, anxiety, and oxidative stress markers. The HIIT group exhibited reduced malondialdehyde (MDA) levels, increased antioxidant enzyme activity, and decreased neuronal damage, displaying that exercise facilitates PTSD-induced impairments by oxidative stress modulation. Besides, Alzoubi et al. (2018) utilized the SPS model to showcase that antioxidants such as Tempol can stop memory impairments related to PTSD by restoring the balance of oxidative stress markers in the hippocampus. This finding corresponds with animal studies on the protective influences of exercise on oxidative stress. In rats, exercise has been demonstrated to boost antioxidant defenses, like catalase and superoxide dismutase (SOD), which are crucial for reducing the detrimental influences of ROS accumulation in PTSD.

The major difference between animal and human studies exists in the complexity and applicability of findings. In humans, the multifaceted nature of PTSD—encompassing psychological, physiological, and behavioral dimensions—makes it challenging to attribute symptom improvements solely to exercise interventions. While human studies demonstrate positive trends in reducing PTSD symptoms by exercise, they also uncover the limitations of exercise in hindering broader lifestyle factors, like diet, or the complexities of comorbid conditions. In animal models, nevertheless, the ability to control variables allows for a clearer understanding of the biological pathways included in PTSD and the potential for exercise to regulate oxidative stress, inflammation, and neuronal plasticity. Animal studies consistently demonstrate that exercise alleviates oxidative damage, enhances neuroplasticity, and protects against cognitive and physiological impairments, which are hallmark features of PTSD. Besides, the findings from animal studies often highlight the specific physiological changes (e.g., reduced inflammation, increased antioxidant enzyme activity) that go along with exercise-induced symptom relief. These mechanisms are not always totaly elucidated in human studies, where improvements are commonly demonstrated in more global terms, like reductions in anxiety or depressive symptoms, without detailed exploration of the biological pathways involved.

Despite the pleasant results from both animal and human studies, divergent findings show that the efficaciousness of exercise in treating PTSD may rely on several factors, comprising exercise type, intensity, duration, and the presence of comorbidities. While animal models often showcase consistent improvements in physiological markers, such as oxidative stress and neuronal damage, human studies uncover more variability in outcomes, with some individuals responding better to exercise than others. Future research should aim to bridge this gap by carring out longitudinal studies that evaluate the long-term impact of exercise on PTSD symptoms, as well as by integrating advanced biomarker analyses to better understand the biological mechanisms at play. In addition, a more nuanced comparison between exercise interventions and other therapeutic strategies, such as pharmacotherapy or psychotherapy, is needed to determine the most efficacious and feasible treatment protocols for PTSD.

To sum up, while both animal models and human studies underscore the potential of exercise as a therapeutic intervention for PTSD, more detailed and comparative studies are needed to refine exercise protocols and understand the interactions between exercise-induced physiological changes and psychological symptom improvement. By synthesizing findings from both domains, we can come up with more targeted, evidence-based interventions for PTSD treatment. Table 1 displays Summary of Exercise Interventions in PTSD Research: Comparative Insights from Animal Models and Human Studies.

**TABLE 1:** Summary of exercise interventions in PTSD research: comparative insights from animal models and human studies.

Authors	Published year	Study type	Exercise intervention	Findings	Significance
Browne, J., et al.	2021	Clinical Trial (Pilot Study)	12-week supervised exercise (Experimental: exercise; Control: wait-list)	Exercise did not improve diet quality, but had positive influences on health	Highlights the challenge of combining exercise and diet interventions for PTSD in older veterans
Hall, K. S., et al.	2020	Clinical Trial (Pilot Study)	Exercise training (Experimental: supervised exercise; Control: wait-list)	Exercise training significantly improved PTSD symptoms, depression, and sleep quality	Demonstrates that exercise can be an efficacious, feasible intervention for PTSD-related conditions in older veterans
Powers, M. B., et al.	2015	Clinical Trial (Pilot Study)	Exercise Augmentation of Exposure Therapy (Experimental: exercise + exposure; Control: exposure only)	Exercise augmented exposure therapy for PTSD showed improvements in PTSD symptoms and BDNF.	Provides support for exercise augmentation in exposure therapy, but calls for more targeted approaches
Young-McCaughan, S., et al.	2022	Clinical Trial (Pilot Study)	Aerobic exercise (Experimental: exercise + imaginal exposure; Control: imaginal exposure only)	Aerobic exercise did not enhance exposure therapy, but improved PTSD symptoms	Suggests that aerobic exercise alone may not enhance exposure therapy efficaciousness in treating PTSD.
Manukhina, E. B., et al.	2021	Animal Model (Experimental PTSD)	HIIT (Experimental: HIIT; Control: sedentary)	Exercise improved PTSD-induced impairments by reducing oxidative stress and neuronal damage	Shows the potential of exercise in animal models to reduce PTSD-induced oxidative stress and cognitive impairments
Koyuncuoğlu, T., et al.	2021	Animal Model (Experimental PTSD)	HIIT (Experimental: HIIT; Control: sedentary)	HIIT reduced anxiety, cognitive decline, and oxidative stress markers, improving antioxidant capacity	Indicates that HIIT could be more efficacious than moderate intensity exercise in reducing PTSD-related impairments
Alzoubi, K. H., et al.	2018	Animal Model (Experimental PTSD)	Tempol (Experimental: Tempol; Control: vehicle)	Tempol prevented memory impairment and oxidative stress in PTSD rats	Suggests that antioxidants may play a role in alleviating PTSD-induced cognitive impairments

## 4 The relationship between the hypothalamic-pituitary-adrenal axis (HPA axis) and PTSD

### 4.1 The role of the HPA axis in PTSD

**FIGURE 2:**
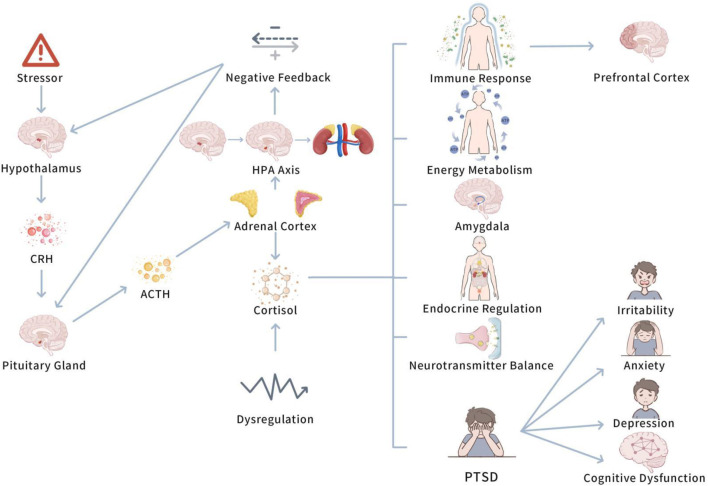
Mechanism of the HPA axis in PTSD.

Before investigating the role of the HPA axis in PTSD, it is significant to understand the basic function of the axis and how it regulates the stress response in normal physiological states. Understanding the basic function of the HPA axis is vital to understanding its dysregulation in PTSD and its far-reaching consequences. Figure 2 *displays mechanism of the HPA axis in PTSD*.

This figure depicts the dysregulation of the HPA axis in PTSD, highlighting the altered cortisol secretion patterns due to impaired negative feedback. Chronic stress leads to prolonged cortisol elevation, contributing to PTSD symptoms, such as anxiety and hyperarousal. Exercise is shown to restore HPA axis balance, stabilizing cortisol levels and reducing stress-induced disturbances.

#### 4.1.1 Basic function of the HPA axis

When an individual confronts a stressor, the hypothalamic-pituitary-adrenal (HPA) axis initiates a stress response by a range of endocrine feedback mechanisms (Kageyama et al., 2021). In particular, the stress stimulus initially activates the hypothalamus to release corticotropin-releasing hormone (CRH), which in turn stimulates the pituitary gland to secrete adrenocorticotropic hormone (ACTH) (Kageyama et al., 2021). ACTH enters the bloodstream and plays on the adrenal cortex, boosting the secretion of cortisol and other hormones. These hormones regulate energy metabolism, immune responses, and other physiological functions, helping the body deal with the stressor (Evanson et al., 2010). The generation of CRH boosts the secretion of ACTH by the pituitary gland, further enhancing the adrenal cortex to release hormones, such as cortisol, which are vital stress hormones that contribute to regulate endocrine, metabolic, and immune processes in the body (O'Connor et al., 2000).

The function of the HPA axis is not limited to activating the stress response; it also involves sustaining system homeostasis by negative feedback mechanisms (Joseph and Whirledge, 2017). When cortisol levels in the body reach a certain threshold, cortisol restricts the release of CRH from the hypothalamus and ACTH from the pituitary gland, thus lowering its own secretion and stopping excessive stress responses. This negative feedback regulation is vital for sustaining endocrine system balance and preventing overactive stress responses (Kageyama et al., 2021).

All in all, the HPA axis activates the stress response by releasing CRH from the hypothalamus, which stimulates the pituitary to secrete ACTH. ACTH triggers the adrenal cortex to release cortisol and other hormones, aiding in energy metabolism, immune responses, and stress adaptation. Cortisol also regulates the HPA axis through negative feedback, retarding CRH and ACTH release to maintain system balance and prevent excessive stress responses.

#### 4.1.2 HPA axis dysfunction and PTSD

Though the HPA axis maintains the body’s endocrine balance by precise negative feedback mechanisms, dysfunction in this system can lead to severe physiological and psychological problems (DeMorrow, 2018). PTSD patients generally exhibit dysregulation of HPA axis function, commonly acted as abnormal elevations or reductions in cortisol levels. Study has demonstrated that cortisol secretion responses in PTSD patients may be excessive or insufficient (Yehuda, 2001). This abnormal cortisol level impacts neurotransmitter balance, immune function, and the function of brain regions related to emotion regulation, memory formation, and fear responses, such as the prefrontal cortex and amygdala (Evanson et al., 2010).

Abnormal cortisol secretion influences various physiological processes, comprising neurotransmitter balance and immune responses, and these changes may deepen emotional symptoms in PTSD patients, such as irritability, anxiety, and depression (Knezevic et al., 2023). At the same time, abnormal cortisol secretion affects multiple physiological processes, comprising neurotransmitter balance and immune responses, which may deepen emotional symptoms in PTSD patients, such as irritability, anxiety, and depression (Knezevic et al., 2023). Besides, prolonged high or low cortisol levels may cause irreversible damage to the nervous system, triggering cognitive dysfunction (Farzi et al., 2015). Moreover, prolonged exposure to stressors can cause HPA axis dysregulation, triggering abnormal cortisol secretion, which affects brain structure and function, especially in the prefrontal cortex and amygdala, thus lowering emotional and cognitive hinderments in PTSD patients (James et al., 2023).

To sum up, dysregulation of the HPA axis function plays a vital role in the pathophysiology of PTSD. Abnormal elevations or reductions in cortisol levels not only influence emotions and behaviors, but may also trigger long-term changes in brain function, further deepening PTSD symptoms.

### 4.2 The regulatory influences of exercise on the HPA axis

**FIGURE 3:**
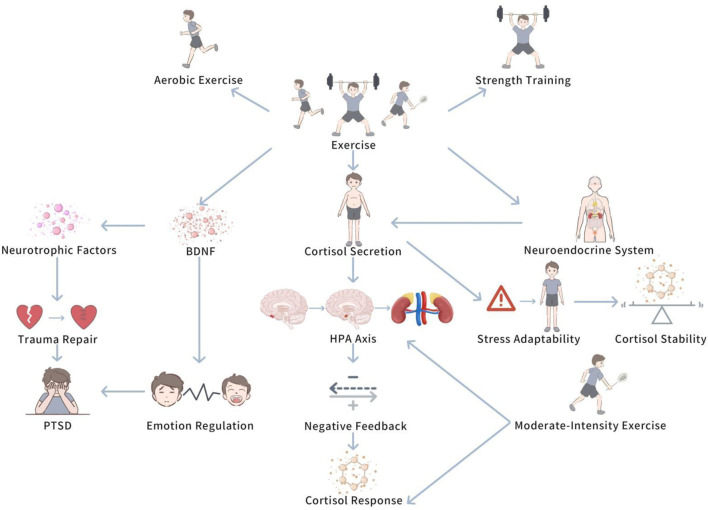
Modulation of the HPA axis by exercise.

Exercise, particularly training of different types and intensities, has a multilevel influence on cortisol levels. This influence encompasses both the modulation of short-term stress responses and the long-term shaping of an individual’s adaptive ability. By understanding the specific mechanisms by which exercise influences cortisol secretion, we are better able to accurately understand the key role that exercise interventions play in PTSD patients. Figure 3 *demonstrates The modulation influence of exercise on HPA axis.*

This diagram demonstrates how exercise, particularly moderate-intensity aerobic exercise, enhances the negative feedback mechanisms of the HPA axis, leading to improved cortisol regulation. The figure also illustrates the potential role of exercise in restoring normal diurnal cortisol rhythms and enhancing stress resilience in PTSD patients.

#### 4.2.1 The impact of exercise on cortisol secretion

Exercise, especially aerobic exercise and strength training, has been demonstrated to regulate the function of the HPA axis, helping PTSD patients restore balance in stress responses (Molina-Hidalgo et al., 2023). Regular exercise can boost cortisol secretion patterns, allowing individuals to gradually adapt to pressure and thus slow down excessive cortisol responses (Hegberg et al., 2019a). For instance, in the initial stress response, exercise may trigger a transient increase in cortisol levels, but with continued exercise, individuals’ stress adaptability gradually enhances, and cortisol responses become more stable (Hegberg et al., 2019a). Moreover, it is worth noting that this phenomenon suggests that exercise not only improve physical health, but also helps PTSD patients restore normal HPA axis function, lowering excessive or insufficient cortisol secretion, thus strengthen the ability to regulate stress responses (Hackney and Walz, 2013).

#### 4.2.2 The impact of exercise on the HPA axis feedback mechanism

Aerobic exercise and strength training can not only boost cortisol secretion patterns, but also improve the HPA axis’s negative feedback mechanisms, thus facilitating normal cortisol cycling and preventing excessive activation of stress responses (Moyers and Hagger, 2023). Study has demonstrated that moderate-intensity aerobic exercise helps boost ance the responsiveness of the HPA axis and stimulates prompt recovery of cortisol levels (Do et al., 2021). Chronic stress states commonly trigger HPA axis dysfunction, displayed as excessive or persistently low cortisol secretion (Zhu et al., 2014). In such cases, regular exercise can restore normal diurnal rhythms of cortisol and enhance negative feedback regulatory functions, lowering the impact of stress on the neuroendocrine system (Moyers and Hagger, 2023).

#### 4.2.3 The role of exercise in post-trauma repair

In addition to regulating the immediate response of the HPA axis, exercise can also play an vital role in the long-term repair process following traumatic experiences (Wang et al., 2023). PTSD patients commonly experience persistent neuroendocrine system imbalances (Vidović et al., 2009). Exercise stimulates the expression of neurotrophic factors, helping to fix the nervous system and restore normal HPA axis function (Björkman and Ekblom, 2022). For instance, exercise can raise levels of brain-derived neurotrophic factor (BDNF), which not only aids in the recovery of neural cells, but also boosts the connectivity between brain regions, thus improving emotion regulation and stress coping abilities (Nowacka-Chmielewska et al., 2022). These research findings prove that exercise not only boosts physical fitness, but also, by promoting the levels of neurotrophic factors, helps PTSD patients recover from trauma-induced damage to the nervous system, thus boosting the restoration of the HPA axis and slowing down the long-term neuroendocrine influences of post-traumatic stress responses.

## 5 The crosstalk between oxidative stress and the HPA axis

### 5.1 The interaction between oxidative stress and the HPA axis

**FIGURE 4:**
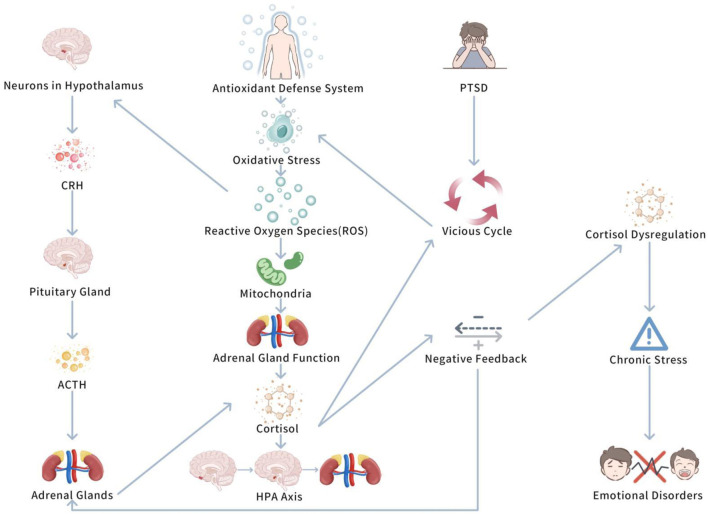
Interaction between oxidative stress and the HPA axis in PTSD.

The interaction between oxidative stress and the hypothalamic-pituitary-adrenal (HPA) axis is a complicated physiological process. Together, they regulate the body’s stress response and play vital roles in the occurrence and progression of Post Traumatic Stress Disorder (PTSD) (Spiers et al., 2015). Oxidative stress, especially the excessive accumulation of reactive oxygen species (ROS), is seen as a key biological marker of PTSD, it not only affects the health of the nervous system, but also promotes PTSD symptoms by regulating HPA axis function (Spiers et al., 2015). Therefore, the interaction between oxidative stress and the HPA axis has become an vital focus in investigating the biological basis of PTSD. Figure 4 *shows Interaction between oxidative stress and HPA axis*.

This figure highlights the bidirectional relationship between oxidative stress and HPA axis dysregulation in PTSD. Chronic stress activates the HPA axis, leading to increased oxidative stress, which further deepens HPA axis dysfunction. Exercise is shown to break this cycle by reducing oxidative stress and normalizing cortisol levels, thus improving PTSD symptoms.

#### 5.1.1 The impact of ROS on the various stages of the HPA axis

Oxidative stress influences the HPA axis in multiple aspects. It is fun that ROS can act on neurons in the hypothalamus, possibly modifying the secretion of corticotropin-releasing hormone (CRH) (Trifunovic et al., 2021). CRH is a key hormone triggering the stress response, which plays on the pituitary to stimulate the secretion of adrenocorticotropic hormone (ACTH), thus promoting the adrenal glands to secrete cortisol and initiating systemic stress responses (Raise-Abdullahi et al., 2023). In PTSD patients, excessive oxidative stress may enhance the secretion of CRH, causing overactivation of the HPA axis and causing abnormal elevations in cortisol (Wilson et al., 2013). These abnormal cortisol elevations are closely linked to the symptom presentation of PTSD.

Moreover, ROS may influence the function of the pituitary and adrenal glands, especially the mitochondria in the adrenal glands (Diebold and Chandel, 2016). Mitochondria are a core source of ROS, and chronic oxidative stress can damage these cells, thus lowering their capacity to secrete cortisol (Lushchak et al., 2023). This process may lead to dysregulation of the HPA axis’s negative feedback mechanism, triggering cortisol secretion disorders, further promoting stress responses and emotional disorders (Kmita et al., 2023). This shows that in PTSD patients, oxidative stress not only changes the activation pattern of the HPA axis, but may also interrupt adrenal gland function, further stimulating a vicious cycle of stress responses.

To sum up, oxidative stress influences the HPA axis in PTSD by augmenting CRH secretion, causing overactivation of the axis and abnormal cortisol levels. ROS can harm pituitary and adrenal gland mitochondria, impairing cortisol secretion and disrupting the HPA axis’s negative feedback, which boosts stress and emotional disorders. This develops a vicious cycle of heightened stress responses.

#### 5.1.2 The bidirectional regulatory relationship between oxidative stress and the HPA axis feedback mechanism

The interaction between oxidative stress and the HPA axis is a bidirectional regulatory process. Oxidative stress not only boosts HPA axis activity, causing cortisol levels to rise, but also stimulates the antioxidant defense system to help mitigate oxidative damage (Spiers et al., 2015). Nevertheless, prolonged cortisol elevation may impair the antioxidant defense system, leading to increased oxidative stress and developing a vicious cycle (Raise-Abdullahi et al., 2023). This bidirectional regulatory relationship is quite obvious in PTSD. Study has demonstrates that PTSD patients commonly exhibit abnormal patterns of cortisol secretion, in particular impaired negative feedback mechanisms, triggering an inability to suppress stress responses (Sriram et al., 2012). Further research has demonstrated that this dysregulation of the negative feedback mechanism plays a vital role in PTSD, potentially being a key factor causing the persistence of chronic stress states (Sriram et al., 2012). This mechanism’s dysregulation not only deepens PTSD symptoms, but may also promote the formation of chronic stress responses, further lowering the patients’ conditions.

In conclusion, oxidative stress and the HPA axis interact in a bidirectional manner, where oxidative stress increases HPA axis activity and cortisol, while also activating antioxidant defenses. Nevertheless, prolonged cortisol elevation can impair these defenses, developing ng a vicious cycle, typically evident in PTSD. In PTSD, abnormal cortisol secretion and weakened negative feedback cause persistent chronic stress and worsened symptoms. This dysregulation may be a core factor in sustaining chronic stress in PTSD patients.

### 5.2 How exercise meanwhile influences oxidative stress and the HPA axis

**FIGURE 5:**
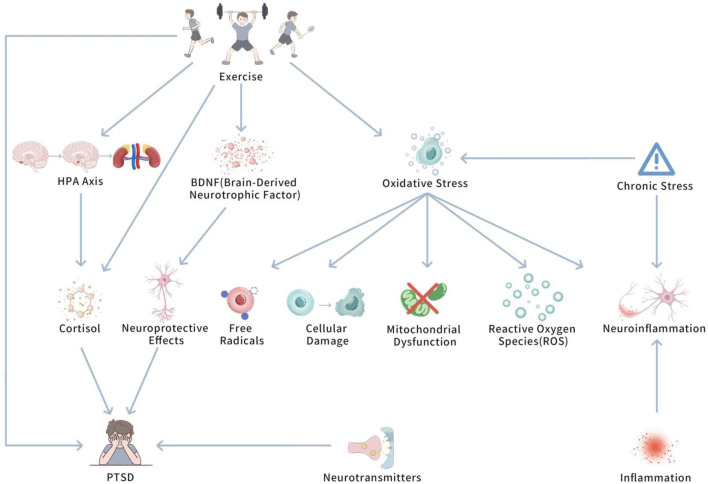
Exercise-induced regulation of oxidative stress and the HPA axis in PTSD.

Exercise has been completely demonstrated to regulate the functions of both oxidative stress and the hypothalamic-pituitary-adrenal (HPA) axis by multiple mechanisms, thus affecting the symptoms of Post Traumatic Stress Disorder (PTSD) patients. Regular exercise can lower the accumulation of reactive oxygen species (ROS) and boost the negative feedback mechanism of the HPA axis, restoring normal stress responses. Figure 5 *depicts Exercise-Induced Regulation of Oxidative Stress and HPA Axis in PTSD.*

This diagram illustrates the mechanisms by which exercise regulates both oxidative stress and HPA axis function in PTSD. It shows how exercise reduces ROS and enhances antioxidant enzyme activity, while simultaneously stabilizing cortisol levels and promoting neural plasticity, ultimately leading to improved psychological and physiological resilience in PTSD patients.

#### 5.2.1 The regulatory role of exercise on oxidative stress

Aerobic exercise (such as running, swimming, etc.) improve cardiorespiratory function and enhances blood circulation, strengthening the efficacious delivery of oxygen and nutrients, thus strengthening the brain and other tissues’ ability to combat oxidative stress (You et al., 2022). Substantial studies have demonstrated that moderate aerobic exercise can vitally improve the activity of antioxidant enzymes in the body, thus strengthening antioxidant defense (Moir et al., 2023; Lesmana et al., 2022). Moreover, higher-intensity exercise (including strength training) can also facilitate the synthesis of antioxidant enzymes, thus lowering exercise-induced oxidative stress (Powers et al., 2022). The generation of oxidative stress is closely linked to the generation of free radicals in the course of muscle contraction (Powers et al., 2022). Nevertheless, appropriate exercise stimulation can activate the cell’s antioxidant defense system, especially by increasing the Nrf2 signaling pathway to improve the expression of antioxidant enzymes (Kawamura and Muraoka, 2018). These series of studies prove that moderate aerobic and strength training not only enhance the body’s antioxidant defenses, but also alleviate the negative effects of oxidative stress on physiological and psychological health by regulating redox balance. Moreover, oxidative stress is induced by the accumulation of ROS (such as hydrogen peroxide and superoxide anion), triggering cellular damage (Kozlov et al., 2024). Moderate exercise can lower oxidative damage induced by chronic stress through raising the activity of antioxidant enzymes and strengthening the body’s antioxidant defense system (Powers et al., 2022). Exercise-induced biological adaptive changes can lower oxidative damage induced by chronic stress, promoting healthy cellular function (Kawamura and Muraoka, 2018).

In a short, aerobic exercise benefits to cardiorespiratory function and blood circulation, boosting oxygen and nutrient delivery to tissues and enhancing antioxidant defenses. Moderate exercise stimulates antioxidant enzyme activity, while higher-intensity exercise (including strength training) also supports antioxidant enzyme synthesis, reducing oxidative stress. Exercise activates the Nrf2 signaling pathway, enhancing antioxidant defense. This helps reduce the negative influences of oxidative stress, boosting both physiological and psychological health by improving redox balance and lowering oxidative damage from chronic stress.

#### 5.2.2 The regulatory role of exercise on the HPA axis

The regulatory role of exercise on the HPA axis function, particularly in chronic stress and post-trauma recovery, has been well recorded. Studies have demonstrated that exercise can obviously boost the negative feedback mechanisms of the HPA axis, regulating cortisol secretion (Hegberg et al., 2019a; Zhu et al., 2022). Particularly, regular aerobic exercise has been found to lower cortisol levels in PTSD patients, thus promoting the adaptability of their stress responses (Hegberg et al., 2019a; Zhu et al., 2022). PTSD patients commonly exhibit lower basal cortisol levels, and this phenomenon is closely linked to HPA axis dysfunction (Nemeroff, 2016). One study have demonstrated that moderate to high-intensity exercise can increase cortisol levels, restoring normal HPA axis function. This influence is related to enhancements in PTSD symptoms (Hackney and Walz, 2013). Especially in the context of chronic stress, exercise can obviously lower the disadvantageous influences of stress on HPA axis function, helping to restore normal fluctuations in cortisol (Hare et al., 2014). By inducing acute cortisol generation and regulating the HPA axis, exercise can boost the adaptability of stress responses and slow down PTSD symptoms (Dong and Lin, 2025). By restoring HPA axis function, exercise improves the body’s ability to adapt to stress, providing new perspectives for the treatment of stress-related disorders.

To sum up, exercise plays a core role in regulating the HPA axis, in particular in chronic stress and PTSD recovery, by boosting negative feedback mechanisms and regulating cortisol levels. Regular aerobic exercise can lower cortisol levels in PTSD patients, restore HPA axis function, and promote stress response adaptability. This strategy offers possibility for treating stress-related disorders by enhancing the body’s ability to manage stress.

#### 5.2.3 The comprehensive influences of exercise

Studies have demonstrated that regular aerobic exercise has a obvious positive regulatory influence on the HPA axis in PTSD patients, this influence restores cortisol levels and lowers stress responses, promoting PTSD patients’ emotional and cognitive functions (Hegberg et al., 2019a; Nowacka-Chmielewska et al., 2022). Particularly, exercise slows down PTSD patients’ overall health by reducing oxidative stress and boosting the normalization of HPA axis negative feedback mechanisms (Hegberg et al., 2019a; Nowacka-Chmielewska et al., 2022).

Further research has displayed that exercise not only strengthens HPA axis function and regulates cortisol levels, but also lowers oxidative impairment and enhances anti-stress influences, helping PTSD patients cope with emotional and psychological problems (Budde et al., 2018). This comprehensive impact is mostly due to the comlicated interaction between exercise-regulated oxidative stress and the neuroendocrine system (Nowacka-Chmielewska et al., 2022). Besides, exercise has obvious influences on promoting physiological symptoms in PTSD patients, especially anxiety and depression symptoms, this is closely related to exercise’s role in increasing the expression of antioxidant enzymes in the body and lowering oxidative damage linked to chronic stress (Nowacka-Chmielewska et al., 2022).

What’s more, in PTSD treatment, exercise may further facilitate its therapeutic influences by regulating the immune system, promoting the secretion of neurotrophic factors (such as BDNF), and anti-inflammatory substances (Sleiman et al., 2016). Current studies have demonstrated that exercise can raise the level of BDNF, thus strengthening neural regeneration and neuroprotective effects (Jaberi and Fahnestock, 2023; Shobeiri et al., 2022). As a main neurotrophic factor, BDNF plays a vital role in the brain’s neural plasticity and remodeling processes, in detail, combining exercise’s antioxidant effects with its promotion of neuroprotective impact can slow down PTSD patients’ psychological symptoms and accelerate neural repair and functional recovery (Calabrese et al., 2014). Moreover, exercise’s regulatory effects on the immune system also show anti-inflammatory effects, lowering inflammation responses caused by chronic stress (Pedersen and Hoffman-Goetz, 2000). Current studies have uncovered that exercise’s regulation of the immune system, by alleviating inflammation responses, plays an obvious role in long-term anti-stress effects (Simpson et al., 2015).

Generally, regular aerobic exercise affects the HPA axis in PTSD patients by restoring cortisol levels, reducing stress responses, and facilitating emotional and cognitive functions. It slows down oxidative stress, strengthens neuroendocrine function, and reduces anxiety and depression symptoms. Exercise also contributes to neuroprotective effects through increased BDNF levels and regulates the immune system, lowering inflammation and helping long-term anti-stress benefits. This combination of antioxidant, neuroprotective, and anti-inflammatory effects accelerates psychological symptom relief and neural repair in PTSD patients.

## 6 Limitations of current evidence and future research directions

### 6.1 Preclinical vs. clinical evidence

One of the most obvious restrictions in the existing literature is the over-reliance on preclinical (animal model) studies compared to clinical (human) studies. While animal studies have been fundamental in elucidating the biological mechanisms underlying PTSD and the impact of exercise on oxidative stress and the HPA axis, their direct applicability to humans continues to be uncertain. The physiological processes in animal models, though highly informative, do not always replicate the complexity of human PTSD, which is affected by a multitude of genetic, environmental, and psychosocial factors. As such, while preclinical evidence supports the hypothesis that exercise can regulate oxidative stress and HPA axis function, the extent to which these findings can be generalized to human populations is still waiting to be investigated. Future research must prioritize large-scale clinical trials with a variety of human populations to confirm the mechanisms observed in animal models. Moreover, randomized controlled trials (RCTs) that focus on comparing the effects of different types of exercise (e.g., aerobic, resistance training, HIIT) on PTSD outcomes, while accounting for comorbidities and baseline physiological differences, will be crucial in applying preclinical findings to clinical practice.

### 6.2 Sample size and study design

Another restricition exists in the comparatively small sample scale and heterogeneity of study designs in clinical trials. Many current studies, especially those in older adult populations with PTSD, include small sample sizes, which may reduce the statistical power and generalizability of findings. Moreover, variations in the duration and intensity of exercise interventions, as well as differences in outcome measures (e.g., PTSD symptom severity, oxidative stress markers, HPA axis function), complicate cross-study comparisons. In addition, the lack of long-term follow-up data limits our understanding of the sustained effects of exercise on PTSD symptoms and underlying physiological processes. To cope with these issues, future studies should aim to standardize exercise protocols, define clear outcome measures, and include larger, more various cohorts. Longitudinal studies will also be vital to evaluate the long-term advantagaes of exercise interventions, including their effects on PTSD symptom recurrence and chronicity. What’s more, examining possible differences in the efficacy of exercise across various PTSD subtypes (e.g., combat-related vs. non-combat-related PTSD) would offer more nuanced comprehension into how exercise might be customized to specific patient populations.

### 6.3 Mechanistic understanding and complex interactions

Although substantial progress has been found in recognizing the core pathways by which exercise may exert therapeutic effects—namely through the regulation of oxidative stress and HPA axis function—our insight of the complicated relationship between these mechanisms continues to be incomplete. Particularly, the bidirectional relationship between oxidative stress and HPA axis dysregulation is still not fully determined. While exercise is hypothesized to alleviate oxidative stress, boost mitochondrial function, and normalize HPA axis activity, the precise molecular and neurobiological pathways involved in these processes require further investigation. In addition, the role of other neurobiological markers, like neuroinflammation and neuroplasticity-related factors like BDNF, in mediating the effects of exercise on PTSD continues to be inadequately explored. Future research should concentrate on dissecting the molecular underpinnings of these mechanisms, potentially incorporating advanced techniques, such as neuroimaging, transcriptomics, and proteomics. The integration of biomarkers into clinical trials will help to recognize the physiological changes associated with exercise interventions and offer a clearer insight of how exercise implays the brain’s stress response system. In addition, studies that combine exercise interventions with other therapeutic modalities, like cognitive-behavioral therapy (CBT) or pharmacotherapy, could elucidate whether exercise boosts the efficaciousness of existing treatments by modulating these biological pathways.

### 6.4 Variability in exercise responses and individual differences

A main challenge in the field of exercise interventions for PTSD is the variability in individual responses to exercise. Factors, such as age, sex, genetic predispositions, baseline fitness levels, and the presence of comorbid conditions (e.g., depression, anxiety, cardiovascular disease) can all affect how individuals respond to exercise. While some studies suggest that exercise leads to substantial improvements in PTSD symptoms, others report minimal effects, particularly in populations with higher levels of comorbidity or more severe PTSD symptoms. To account for this variability, future research should explore personalized exercise programs that consider individual characteristics and response profiles. For instance, the use of machine learning and other data-driven strategies could help identify subgroups of individuals who are more likely to benefit from specific types of exercise interventions. In addition, examining the role of genetic factors in determining exercise efficacy could triger the progression of tailored, precision-based strategies for PTSD treatment.

### 6.5 Integration of exercise with other therapeutic approaches

While exercise is increasingly viewed as a valuable adjunctive treatment for PTSD, its effects may be amplified when combined with other therapeutic strategies. For instance, exercise interventions could be integrated with exposure therapy, mindfulness-based therapies, or pharmacotherapy to boost treatment outcomes. Nevertheless, limited research has explored the synergistic effects of combining exercise with other therapies, and the optimal timing, duration, and sequencing of these interventions remain unclear. Future studies should investigate the potential for exercise to augment existing treatments and whether specific combinations of therapies cause superior outcomes. In addition, understanding the mechanisms by which exercise interplays with psychological therapies and medications will offer important insights into how to optimize treatment plans for individuals with PTSD.

## 7 Conclusion, future research directions, and clinical applications

### 7.1 Conclusion

By our comprehensive review, we have determined that PTSD is a complicated mental health condition with vital physiological and psychological implications, commonly associated with oxidative stress and HPA axis dysregulation. Conventional treatment strategies, while valuable, commonly unable to completely handle the underlying biological mechanisms that cause symptoms of post-traumatic stress disorder. Our review emphasizes the potential of exercise as an adjunct non pharmacological therapy. By promoting the antioxidant defense system, alleviating oxidative stress, and restoring HPA axis function, exercise can reduce emotional and physiological symptoms associated with post traumatic stress disorder. Regular exercise, whether its aerobic exercise, strength training, or HIIT, can boost neural plasticity, promote cognitive recovery, and boost stress recovery ability. Besides, when combined with conventional therapies, such as cognitive-behavioral therapy (CBT) and medication, exercise can improve overall treatment outcomes and offer a comprehensive strategy for managing post traumatic stress disorder.

### 7.2 Future research directions

Although increasing evidence has been helping the therapeutic potential of exercise in the treatment of post traumatic stress disorder, there are still several important issues. Future research should focus on the different effects of various forms of exercise, including aerobic exercise, strength training, and HIIT, on oxidative stress and HPA axis regulation in individuals with post traumatic stress disorder. Understanding the dose-response relationship, such as the optimal exercise intensity, frequency, and duration for patients with post traumatic stress disorder, will contribute to improve exercise programs to reach maximum efficacy. Moreover, more research is required to explore how individual factors, such as genetics, trauma severity, and comorbidities (such as depression or anxiety) affect the efficaciousness of exercise interventions. A tailored exercise program that combines genetic, epigenetic, and psychosocial factors can provide more tailored and efficacious treatment strategies. A longitudinal study assessing the long-term effects of exercise on the recurrence or relapse of post traumatic stress disorder symptoms is also vital for establishing exercise as a preventive measure. At last, integrating advanced neuroimaging and biomarker analysis will offer deeper insights into the neural and endocrine pathways through which exercise affects brain function, emotion regulation, and stress response systems.

### 7.3 Clinical application

The translation and clinical implementation of post-traumatic stress disorder exercise interventions require a precise medical framework that integrates evidence-based protocols, interdisciplinary collaboration, and patient specific adaptations. A multidisciplinary team composed of psychiatrists, exercise physiologists, and rehabilitation experts is vital for designing interventions that handle psychological and physiological mechanisms. For instance, psychiatrists can link exercise patterns with neuroplasticity goals backed by functional MRI studies (such as reducing amygdala hyperactivity through aerobic training), while exercise physiologists personalize their plans based on oxidative stress biomarkers and physical abilities. There is evidence to back stratified exercise doses: moderate intensity aerobic training can reduce the severity of post traumatic stress disorder symptoms and stabilize cortisol rhythms, while HIIT can boost mitochondrial function in preclinical models. Nevertheless, for patients with severe hyperarousal, HIIT needs to be careful to avoid triggering physiological reactions related to trauma. The obstacles faced by vulnerable groups, like the separation of trauma survivors or physical limitations of traumatic brain injury patients, can be alleviated by trauma based adaptations, like yoga or water therapy. Technical tools (like wearable devices for real-time pressure feedback) and remote medical platforms have further improved compliance, particularly in rural areas. Standardization efforts should concertrate on developing guidelines for post traumatic stress disorder, comprising contraindications for HIIT in suicide patients and progression standards for sedentary individuals, while training programs for trauma informed exercise prescriptions are crucial to ensure safety and efficaciousness. This comprehensive approach positions exercise as a critical therapeutic component, utilizing its dual affects on oxidative stress and HPA axis regulation to enhance treatment participation and resilience in post traumatic stress disorder care.
